# The Properties of Concrete Utilizing Partial Aggregate Replacement with Locally Sourced Mediterranean Agro-Waste

**DOI:** 10.3390/ma19112187

**Published:** 2026-05-22

**Authors:** Sandra Juradin, Ivanka Netinger Grubeša, Martina Milat, Vladimir Divić, Dunja Šamec, Dino Rapić

**Affiliations:** 1 Faculty of Civil Engineering, Architecture and Geodesy, University of Split, Matice hrvatske 15, 21000 Split, Croatia; martina.milat@gradst.hr (M.M.); vladimir.divic@gradst.hr (V.D.); 2 Department of Construction, University North, 42000 Varaždin, Croatia; inetinger@unin.hr; 3 Department of Food Technology, University North, 48000 Koprivnica, Croatia; dsamec@unin.hr; 4 Jolos d.o.o., Gundulićeva 26A, 21000 Split, Croatia; dinorapi285@gmail.com

**Keywords:** fruit pits, fruit seeds, agro-concrete, interfacial transition zone (ITZ), concrete properties, ash water

## Abstract

The growth of the global population has led to increased demand for agricultural products, resulting in greater agricultural waste production. One sustainable response to this challenge is using agricultural waste as raw material in building materials. This study examines the potential for partial replacement of natural aggregates in concrete with agricultural waste from typical Mediterranean fruits: sour cherry pits, grape seeds, ground olive pits, and carob seeds. To evaluate the effect of treatment on the behavior of agro-waste aggregates, ground olive pits were used untreated, treated with ash water, or treated with seawater. Carob seed concrete deteriorated during water curing due to seed swelling and tannin-related degradation, revealing its unsuitability without prior stabilization. Partial replacement of natural aggregates with agricultural waste resulted in decreased density, ultrasonic pulse velocity (UPV), dynamic elastic modulus, compressive strength, and thermal conductivity, while increasing saturated water absorption. Treatment with ash water on ground olive pits improved the interfacial transition zone (ITZ), resulting in 29% increase in compressive strength relative to untreated ground olive pits. Concrete with ash water treated ground olive pits demonstrated the highest practical potential among all tested agro-waste concretes.

## 1. Introduction

Global population growth has intensified demand for essential human needs, particularly food and civil infrastructure, which places substantial pressure on both the agricultural and construction sectors. Expanded agricultural activities have led to greater generation of agro-waste, introducing considerable challenges relating to waste management and environmental sustainability. At the same time, the construction and operation of buildings account for approximately 40% of global carbon dioxide emissions, with about 15% of these emissions stemming from the production of construction materials [1]. In Europe, the construction sector is the leading consumer of natural resources, responsible for nearly half of all material extraction and contributing significantly to resource depletion [2]. Globally, resource extraction has surged from 7 gigatons (Gt) in 1900 to 89 Gt in 2015, representing a thirteen-fold increase in the modern industrial period [3].

Addressing these interconnected environmental challenges necessitates innovative strategies, such as the integration of agro-waste into construction products. Utilizing agricultural waste as a raw material not only presents a green solution for waste disposal but also reduces reliance on traditional natural aggregates. Agro-waste, also known as agricultural waste, refers to non-product residues generated during the cultivation and processing of agricultural products of plant or animal origin [4]. Agro-waste is most commonly reused within the same sector in which it is generated, particularly in the food or agronomic sectors, where it serves purposes such as animal feed, compost, soil amendments, or bio-fertilizers. However, a significant portion of agro-waste is non-digestible, chemically complex, or contains compounds that limit its safe or efficient reuse in food or agricultural applications. Because of these constraints, such waste cannot be directly reintegrated into the original sector. As a result, increasing research efforts are focused on exploring alternative uses of agro-waste in other sectors, including energy production, materials science, construction, and biotechnology, with the aim of adding value while reducing environmental impact. Recent research increasingly explores its use in construction materials [5], both for thermal insulation and as structural components [6].

The integration of agro-waste into concrete supports the advancement of circular economy principles, sustainable development, and improved energy efficiency within the construction industry. Moreover, this approach can offer economic advantages by lowering concrete production costs and mitigating environmental impacts [7]. Such benefits are increasingly critical, given that concrete is the world’s second most widely used substance after water, with annual consumption approaching 30 billion tons [8,9]. Agro-waste can be incorporated into concrete in the form of a powdery substance as a substitute for cement or in the form of an aggregate in which it replaces fine and coarse mineral aggregate. Some examples of cementitious substitute materials are rice husk ash, rice straw ash, bamboo leaf ash, sugarcane ash, corn cob ash, coconut husk ash, wheat straw ash, elephant grass ash, etc. [10,11,12,13,14,15,16], which are all derived from lignocellulose agricultural residues that are inherently rich in cellulose, hemicellulose, lignin, and especially silica. Their common chemical characteristics, a high content of amorphous silica and other reactive oxides, makes them particularly valuable in non-food applications, where they generally exhibit excellent pozzolanic properties, enabling their use in construction materials and cement replacement. Common examples of agro-waste applied in concrete production as aggregate include rice husk [5,17,18,19,20], corn cob [21,22,23,24,25,26,27,28,29], wheat straw [30], coconut shell [31,32,33,34,35,36,37,38,39,40], palm kernel shell [41,42,43,44,45,46,47,48,49,50], hazelnut, peanuts, pistachios shells [51,52,53,54,55], and fruit pits such as peaches, nectarine, and apricot [56,57,58,59,60,61,62]. The agro-aggregates consist of organic waste and are subject to different levels of biological decomposition when exposed to an aggressive environment such as cement composites. One potential solution to this issue is the implementation of technological treatment processes. As stated in references [63,64], the pre-treatment of oil palm shell (OPS) with hot water significantly improves the removal of the oily surface from the shells and increases the bond between the cement paste and the OPS, leading to enhanced mechanical properties. To minimize chemical incompatibility between cement and plant biomass, sugarcane bagasse particles were immersed in cold water for 24 h, whereas coffee husk particles were soaked overnight in water and a 1% NaOH solution for 24 h, followed by washing in cold water [65]. Hydrophobic surface treatments were applied to coconut shell, oil palm shell, and areca nut shell; when reinforced with natural fibers, these offer a viable pathway to environmentally sustainable lightweight concrete that boasts enhanced durability and a lower carbon footprint [66]. Utilizing agricultural waste as aggregates in concrete mixtures primarily enhances thermal and acoustic insulation properties, decreases the density and overall weight of the concrete, and consequently leads to economic advantages. Agricultural waste in fresh concrete can both increase and decrease workability, while in hardened concrete, this mainly reduces its mechanical characteristics, i.e., compressive and tensile strength [67].

To extend current research in this area, the present study focuses on aggregate replacement using pits from four locally sourced Mediterranean fruits: sour cherry, grape, olive, and carob. The production of this fruit will be presented below, as well as previous examples of their application in cement composites.

## 2. Production and Current Application of Mediterranean Agro-Waste

**Figure 1:**
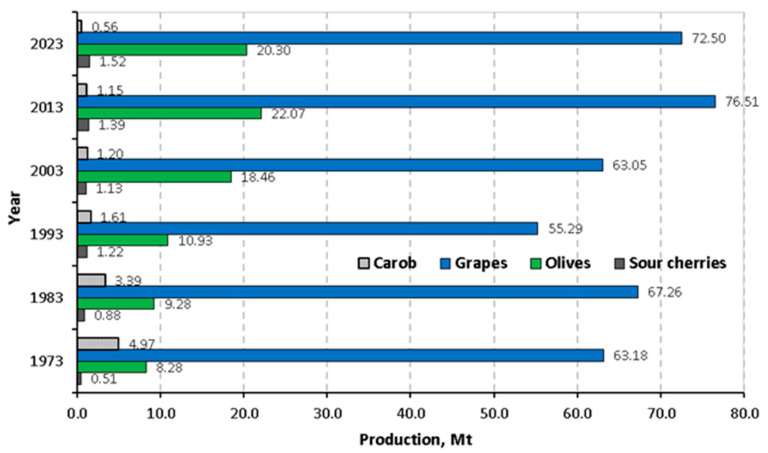
Production of sour cherry, grape, olive and carob in period from 1973 to 2023 according to Food and Agriculture Organization of the United Nations (FAO) [69].

**Sour cherry** (*Prunus cerasus* L.) is one of the most popular fruits, commonly enjoyed fresh and in processed forms [68]. According to Figure 1, in the last 50 years, their production has tripled and in 2023 reached a production of 1.52 Mt [69]. Approximately 85% of cherries are processed, and 30–40% of the total production is intended for the global fruit juice industry [70]. Processing sour cherries generates a significant amount of waste. Once the cherries are juiced and quickly frozen, the leftover pomace, which includes the skin and flesh, along with the pits, is typically discarded [71]. For instance, in the U.S., a single year’s production can generate about 18.1–22.7 kt of this waste (data for 2016), or approx. 15% of the total production [72]. The sour cherry pit (stone) is primarily composed of a hard, lignocellulosic shell that protects the seed inside. This shell consists mainly of cellulose, hemicellulose, and lignin, which give it high mechanical strength and make it resistant to digestion and degradation. Sour cherry pits are a good candidate for use as cement aggregate because they are lightweight, mechanically robust due to their lignocellulosic structure, and have a rough surface that promotes good bonding with the cement matrix. In addition, their chemical stability and status as an agricultural waste make them a sustainable alternative to conventional aggregates. Netinger Grubeša et al. [67] completely replaced the 4–8 mm dolomite aggregate fraction in concrete with cherry sour pits that were either untreated or treated with 2.5 or 5% NaOH. The treatment increased water absorption and the appearance of the pits’ surface, and the concretes with replacement, regardless of the treatment, had lower density, thermal conductivity coefficient, and compressive strength. Bhujel et al. [73] investigated the replacement of natural coarse aggregate with *Prunus cerasoides* shells at varying proportions ranging from 0% to 40% by weight. Their findings indicated a consistent reduction in density, mechanical strengths, and elastic modulus as the proportion of *Prunus cerasoides* shells increased. Conversely, water absorption exhibited a steady rise with higher shell content. Comparable compressive strengths for M20 grade concrete were achieved with 10% and 20% *Prunus cerasoides* shell replacement.

**Olives** (*Olea europaea*): Globally, 64.8% of olive production occurs in Europe, with Spain, Italy, and Greece being the leading producers, all located in the Mediterranean region. Annually, over 20.3 Mt of olives are harvested worldwide, which is a 2.5-fold increase compared to 1973 [69]. Olive stones make up a notable portion of the overall weight of this fruit (20–28%) and are regarded as a by-product in the processes of olive oil extraction and the production of pitted table olives [74]. According to [75], for every 100 kg of processed olives, about 30 kg of pits are obtained, which depends on the variety and quality of the fruit. Olive pits, similarly to cherry pits stones, are primarily composed of a hard lignocellulosic structure rich in cellulose, hemicellulose, and especially lignin, along with small amounts of inorganic minerals. This composition makes them non-digestible and unsuitable for food or feed applications, as the rigid structure resists biological breakdown and offers no nutritional value. Owing to their hardness, low density, and rough surface texture, olive stones are therefore better suited for alternative uses. This agricultural waste is used for fuel because it is cheaper than pellets, and has the same function as pellets, or is disposed of in landfills or dumped somewhere in nature, together with the pomace. Ferreiro-Cabello et al. [74] tried to develop a lightweight mortar that includes olive stone residue (in varying percentages), without the need for supplementary treatments. The authors concluded that an aggregate replacement of 0–30% could be accomplished, with the highest replacement of ground olive stones (30%) resulting in a 15% reduction in density compared to the reference mortar. The compressive strength for the various doses examined remained above 70% of the strength of the reference mortar; however, the flexural performance was significantly compromised, exhibiting values of approximately 50%. EL Boukhari et al. [76] used two types of olive waste (olive pomace solid aggregates and olive pomace solid aggregates immersed in olive mill wastewater) as a partial substitute (0–15%) for natural sand in structural concrete. The concrete specimens containing 5% olive pomace solid aggregates and immersed in olive mill wastewater in a dry condition exhibited the closest mechanical properties to the reference concrete. Moreover, the inclusion of olive waste improved the thermal conductivity of the concrete. Del Río Merino et al. [77] tested three types of olive stones (whole pit, crushed, and calcined) as sustainable substitutes for expanded clay aggregates. The authors determined that substituting expanded clay only with calcined olive stones is economically viable in the manufacturing of lightweight mortars, which can reach densities that are up to 30% lower than those achieved with lightweight mortars made from expanded clay. Beyond testing mechanical, thermal, and economic aspects, another study [78] assessed the environmental implications of incorporating ground olive stone as a partial replacement for natural fine aggregate in 1 m^3^ of mortar at various replacement levels, using a Life Cycle Assessment (LCA) methodology.

**Grapes** (*Vitis vinifera*): The cultivation of grapes is of significant global economic relevance due to their extensive use for various purposes, such as fresh consumption, wine production, raisins, juices, and other products [79]. The production of grapes in 2023 amounted to 72.5 Mt, and according to Figure 1, it can be seen that grapes have seen continuous production. Wine production in Europe accounts for 71.4% of production over the past 50 years, and the three countries with the largest wine production are Italy, France and Spain. The wine processing sector produces a notable amount of waste, which constitutes approximately 13.5–14.5% of the total production. The primary waste components derived from grapes include pomace, seeds, and stems. Grape seeds generally account for 2–5% of the overall grape mass and are responsible for approximately 38–52% of the solid waste generated by the wine industry [79,80]. Grape seeds are widely used in the food, cosmetic, and pharmaceutical industries, mainly for the extraction of grape seed oil and polyphenol-rich antioxidants due to their high content of lipids and bioactive compounds. After oil extraction, the remaining solid residue has limited value for food applications because of its rigid lignocellulosic structure and low digestibility. In [67] fine dolomite aggregate was substituted with three types of grape seeds—untreated and pre-treated with 2.5% or 5% NaOH solution. The substitution of fine aggregate with grape seeds led to a decrease in the workability of the concrete, while concrete with untreated grape seeds achieved 10% of the compressive strength, 25% of the thermal conductivity coefficient and 71% of the density compared to the referent concrete. The concrete treated with grape seeds disintegrated, probably due to the elevated tannin content present in the grape seeds. In [81] four groups of aggregates were examined: one group utilized the complete grape pomace, two additional groups employed stalks and skins individually, and a final group consisted of coarsely ground stalks and found that grape by-products appear to exhibit favorable thermal, mechanical, and acoustic properties, making them suitable for use as insulation materials in the construction of public buildings.

**Carob** (*Ceratonia siliqua* L.): This is an evergreen Mediterranean plant whose history dates back to ancient Egypt, where it was used as livestock feed. In Arab culture, carob seeds served as a unit of weight, known as a qirat or karat; this standard weight later became the yardstick for measuring gold and precious stones [82]. In the observed period 1973–2023, carob production fell to 11% of the 1973 production, when it amounted to 4.97 Mt (Figure 1). Carob is primarily utilized in food and pharmaceutical industries for locust bean gum extraction, which is valued as a thickening and stabilizing agent [83]. The remaining seed shells are non-digestible and low in nutritional value, making them unsuitable for food or feed applications. In total, 90% of the weight of carob pods is pulp and the remaining 10% are seeds (beans), which are extremely hard [84]. In [85] a new biopolymer from locust (carob) bean gum was used as a viscosity-modifying admixture in cement paste. Cement pastes with carob bean gum showed a significant improvement in rheological properties compared to the reference mixture, but it affected the hydration delay of cement mortars. In a study carried out by Clausell et al. [86], it was shown that carob tannin possesses considerable potential as an additive for clay-based construction materials, particularly as a superplasticizer. Research conducted by Zelada et al. [87] examined the impact of partially adding carob ash to a concrete mix, showing that incorporating 6% carob ash enhanced the mechanical properties and reduced the water penetration depth. In contrast, in the present study, carob seeds are used as aggregates in concrete, as the seeds are very hard.

The aim of the paper is to examine the potential of local agro-waste as aggregate in concrete. In addition, due to the availability of ground olive pits, the effect of environmentally friendly pre-treatments on this material are investigated.

## 3. Experimental Part: Material and Method

In the experimental part of the paper, a total of 8 mixtures were made: a referent concrete (RC) with crushed limestone aggregate fractions 0/4 and 4/8 mm, four mixtures in which the fine fraction of the aggregate was replaced with grape seeds (G), untreated ground olive pits (O), ground olive pits treated with ash water (OA), ground olive pits treated with seawater (OS), and three mixtures in which the coarse fraction of the aggregate was replaced with sour cherry pits (SC), whole carob seeds (WC) and hulled carob seeds (HC). Grape seeds were collected from a winery Feričanci, region Slavonija, Croatia, ground olive pits from the oil mill near Split, Croatia, sour cherry pits from Vinka plus d.o.o., Vinkovci, Croatia and carob seeds from Green Captain, obrt za proizvodnju, Suđurađ, Šipan, Croatia.

The treatment of ground olive pits was carried out as follows: Ground olive pits were obtained from local oil mills. The sample was divided into 3 groups: the first was simply washed with tap water, the second was placed in ash water, and the third in seawater. Ash water (Lye) is a powerful alkaline base that is produced by combining water with natural wood ashes. For the purposes of this study, ash water was obtained from water in which sieved ash was boiled and then more clean water was added, so that the final solution was 1.5 kg of ash and 15 L of water. Ground olive pits were placed in this solution for a period of 7 days, and then removed from the solution and washed well in tap water and dried in the sun. The seawater treatment also lasted 7 days and the pits were washed with tap water and left to dry in the sun. Other aggregates were used untreated.

**Figure 2:**
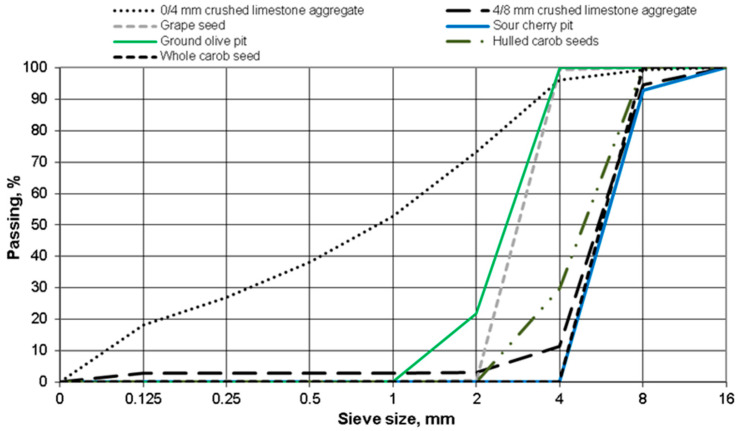
The gradation curves of the aggregates.

**Figure 3:**
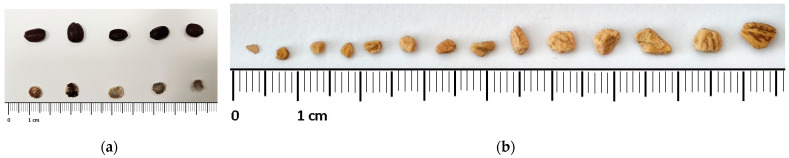
Seeds and pits with metric ruler: (**a**) whole and hulled carob seeds; (**b**) ground olive pits.

The absorption rates of aggregates were determined in accordance with the EN 1097-6 [88] while the gradation curves (Figure 2) were determined in accordance with the EN 933-1 [89]. Carob seeds possess a remarkable hardness and do not readily absorb water [90]; therefore, their water absorption was not measured. Some other characteristics have been determined on the agro-waste. Characteristics of seeds and pits were examined through assessments of bulk density, seed weight, length, and width. Bulk density was measured by filling a 100 mL graduated cylinder with seeds and recording the corresponding mass. Each measurement was conducted three times for accuracy. Bulk density was calculated as the seed mass (g) divided by the volume occupied in the cylinder (cm^3^). Seed weight was determined using an analytical balance, where 20 seeds were weighed, and their average mass per seed (mg) was computed. Measurements of seed length and width were carried out on 20 seeds using a metric ruler with a precision of 0.5 mm, and the average dimensions per seed were derived, as illustrated in Figure 3a. Measuring the length, width and weight of ground olive pit pieces is not applicable (Figure 3b), but the particle size is shown by a gradation curve (Figure 2). The properties and appearance of aggregates are given in Table 1.

For the preparation of concrete specimens, a CEM II/A-LL 42.5 R cement manufactured by Cemex Croatia was used, with a density of 3.08 g/cm^3^ in accordance with EN 197-1 [91], the fineness according to EN 196-6 [92] of 4040 cm^2^/g and 6.4% residue on a 45-micron sieve according to [93]. The concrete mixtures were prepared using a tap water and superplasticizer derived from modified polycarboxylates (MasterGlenium ACE 430 supplied by Master Builders Solutions GmbH - Podružnica Zagreb za trgovinu, Zagreb, Croatia), which has a density of ρ = 1.06 ± 0.02 g/cm^3^, pH = 5.5 ± 1.1 and is dosed with 0.5% of the weight of the cement.

**Figure 4:**
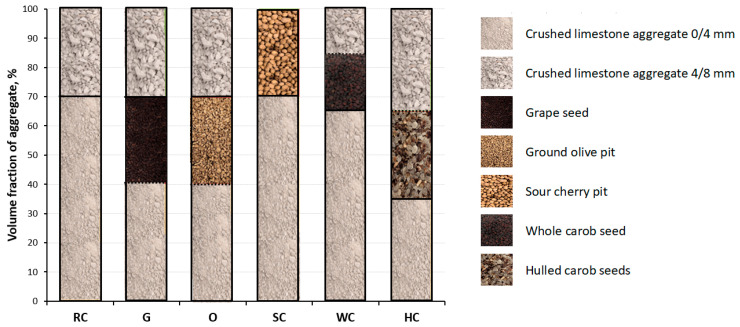
Volume fraction of each aggregate type shown for each mixture.

In the RC, G, SC, O, OA and OS mixtures, the volume fraction of the fine fraction was 70% and coarse aggregate was 30%. The amount of fine aggregate replacement in G, O, OA and OS was 30% by volume, while in the SC the amount of coarse aggregate replacement was 30%. In order to examine the influence of particle size distribution on the workability of concrete in mixtures with carob, the particle size distribution was also varied. In mixtures with hulled carob, the volume fraction of fine aggregate was 35% and coarse aggregate was 65%. In the HC mixture, 30% of coarse aggregate was replaced with hulled carob seeds, while in the WC mixture, the volume fraction of fine aggregate was 65% and coarse aggregate was 35% and the replacement of coarse aggregate was 20%. The reason for this is that when separating the seed from the pulp of the carob, it is much more difficult to obtain whole grains, so a smaller amount was supplied. The volume fraction of each type of aggregate is graphically shown in Figure 4. The composition of each mixture is shown in Table 2.

All specimens were compacted using an immersion vibrator except for specimens G, OS and HC due to poor workability; they were compacted on a vibrating table instead. Specimens were demolded after 24 h and put in the pool with water for another 27 days of water curing.

The consistency of fresh concrete was assessed using the slump test method in compliance with EN 12350–2 [94]. The concrete was cast in 150 mm cubes for testing density (EN 12390-7, [95]), ultrasonic pulse velocity (UPV) (EN 12504-4, [96]), and compressive strength (EN 12390-3, [97]). Dynamic modulus of elasticity (*E_din_*) was determined based on UPV (*v*), presuming a Poisson’s ratio (*µ*) of 0.2, and the density (*ρ*) of hardened concrete in accordance with Equation (1):

(1) $$E_{din}=\frac{v^{2}\rho (1+\mu )(1-2\mu )}{1-\mu },$$

Also the concrete was cast in cylinders of 75 mm diameter and 200 mm height for testing saturated water absorption and sorptivity coefficient S ([98], ASTM C1585-13, [99]), which was determined based on Equation (2):

(2) $$S=\frac{\frac{∆M}{A}}{\sqrt{t}},$$

where Δ*M* is the mass increase in water absorbed by the cylinder surface which was exposed to water, *A* is cross-section of the cylinder, and *t* is time measured in hours (in this case *t* = 24 h). Less than 5 mm of the cylinders were submerged in water, and prior to weighing, any excess surface moisture was carefully removed using a towel.

**Figure 5:**
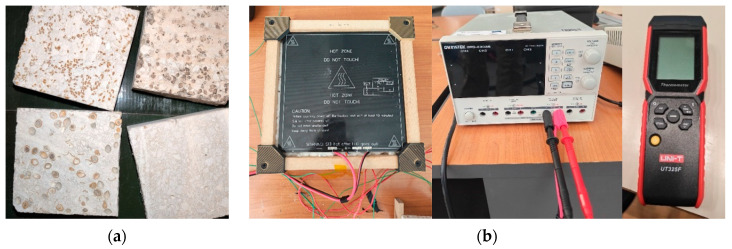
(**a**) Concrete slices cut from 100 mm concrete cubes; (**b**) the experimental set up for thermal conductivity testing.

Thermal conductivity was tested on slices cut from 100 mm concrete cubes, with each slice measuring 100 × 100 × 30 mm (Figure 5a). The testing was performed by applying a controlled heat input to the specimen and monitoring temperature at multiple locations.

Heat was supplied using a flat Printed Circuit Board (PCB) heater with dimensions 220 × 220 mm and a nominal power of 16 W. During testing, the heater was operated at approximately 10 W. The specimen was placed flat on the heating plate, with a thermal couplant applied between the specimen and the heater to improve thermal contact. The bottom side of the PCB heater was insulated using a thermal insulation material (ceramic fiber wool). The top surface around the specimen was left uncovered.

Electrically, the heater behaves as a resistor whose resistance varies with temperature. Therefore, voltage and current were measured and recorded during heating, and the instantaneous electrical power was computed from the measured values. In a simplified analysis, it was assumed that the electrical power delivered to the heater corresponds to the heat flux input to the specimen, proportional to the contact area (i.e., losses to the environment, lateral conduction in the heater, and radiative heat transfer were neglected).

**Figure 6:**
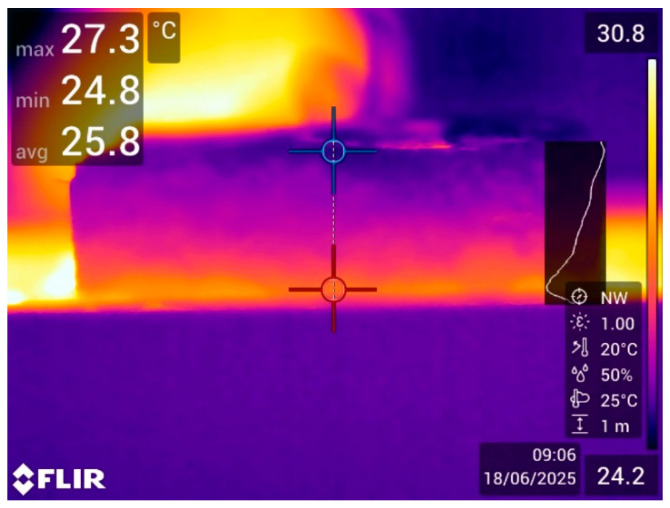
Thermal gradient recorded using thermal camera.

Temperature was measured using type K thermocouples and recorded using a temperature acquisition system (logger). One thermocouple was placed near the heater–specimen interface, while the remaining thermocouples were distributed across the specimen at predefined measurement positions. The test duration was sufficient to capture temperature evolution on all measurement channels. Figure 5b shows the experimental setup. The experiment was also monitored using a thermal camera (FLIR 1020sc 28°) to observe thermal gradients and emissivity variation (Figure 6).

Two approaches were used for data processing. In the simplified approach, measured temperatures were extrapolated to steady state, and thermal conductivity was estimated using the input power, the distances between thermocouples, and the differences between the extrapolated steady-state temperatures. In the more advanced approach, the transient heat conduction equation was solved numerically in MATLAB (MATLAB. Version R2023b, The MathWorks, Inc., Natick, MA, USA, 2023.) for the known specimen geometry and density, using the measured input power curve, while treating thermal conductivity and specific heat capacity as unknown parameters. The numerical model produced time-dependent temperature histories at locations corresponding to the thermocouple positions. An optimization problem was then formulated to identify thermal conductivity and specific heat capacity by minimizing the deviation between the modeled and measured temperatures. Both approaches yielded results with a maximum relative difference of up to 4% (with respect to the advanced model). Results from the simplified model were used further in this analysis.

## 4. Results and Discussion

The measured slump results are given in Table 3 and standardized consistence classes are determined according to EN 206 [100]. The concrete mixtures were prepared in a laboratory mixer. When preparing mixtures RC, G, SC and OS, extra water was added to the drum to be absorbed by the aggregate, while for mixtures O and OA, the aggregate was previously saturated and then added to the drum. Substituting the fine particles of crushed limestone with grape and olive pits in mixtures G and OS led to a notable decrease in the consistency of the concrete while pre-soaking had a positive effect on the consistency of the O and OA concretes. This pre-saturation process helps reduce excessive water absorption during the mixing phase, as the authors concluded in [63,101]. The SC mixture has better workability compared to the reference concrete, and the reason for this is probably due to the completely round pits and, according to Figure 2, the almost ideal granulometric curve of 4/8 mm. WC and HC have lower workability but also different particle size distribution. The HC mixture had a higher proportion of coarse aggregate and the carob seeds are flatter than WC, so the workability is lower.

**Figure 7:**
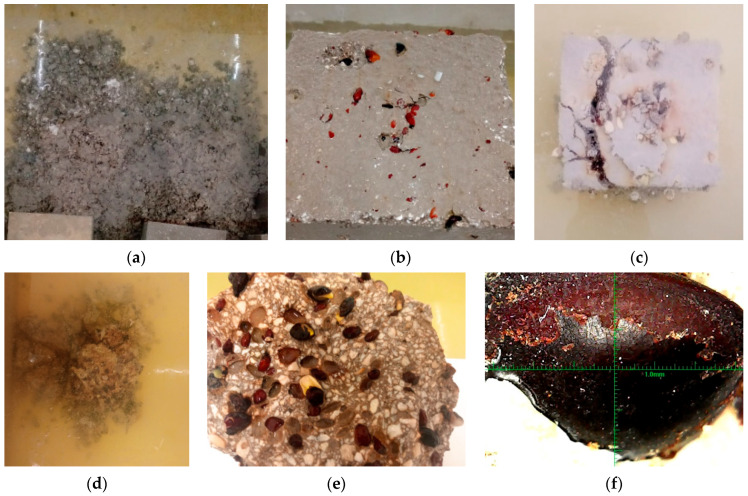
Disintegration of carob concrete: (**a**) HC mixture; (**b**) spalling of WC concrete; (**c**) cracking of WC concrete; (**d**) disintegration of WC concrete; (**e**) remaining part of WC cube; (**f**) microscopic image of carob seed in concrete.

The above mentioned tests were performed in the hardened state of the concrete, at an age of 28 days. It was observed that the specimens with carob HC and WC disintegrated during water curing. The HC mixture disintegrated relatively quickly after being placed in water because it had a higher carob content and hulled seeds (Figure 7a), while the WC mixture disintegrated gradually; see Figure 7b–f. This disintegration is probably attributed to seed swelling and high tannin levels, as carob seeds are rich in tannins, polyphenols, and flavonoids, but carob seeds also serve as natural alternative to synthetic thickeners [83]. Because carob seeds have a hard and impermeable skin, they need some external influence to get water into the center of the seed. In the case of HC specimens, this was not a problem because the seeds were hulled, but for WC specimens this took much longer even though whole seeds were in an alkaline environment. There are studies that have examined swelling plant base aggregates and how pre-treatments affect this, but these are mostly materials based on cellulose, hemicellulose and lignin [102]. Concrete disintegration due to the action of tannins was also observed in the study [67].

**Figure 8:**
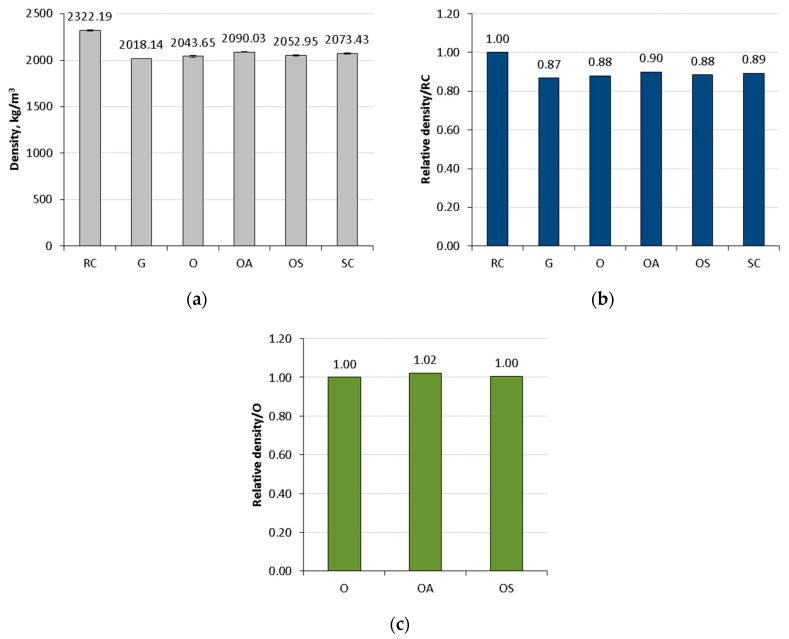
Density of concrete specimens: (**a**) absolute values; (**b**) relative values/RC; (**c**) relative values/O.

**Figure 9:**
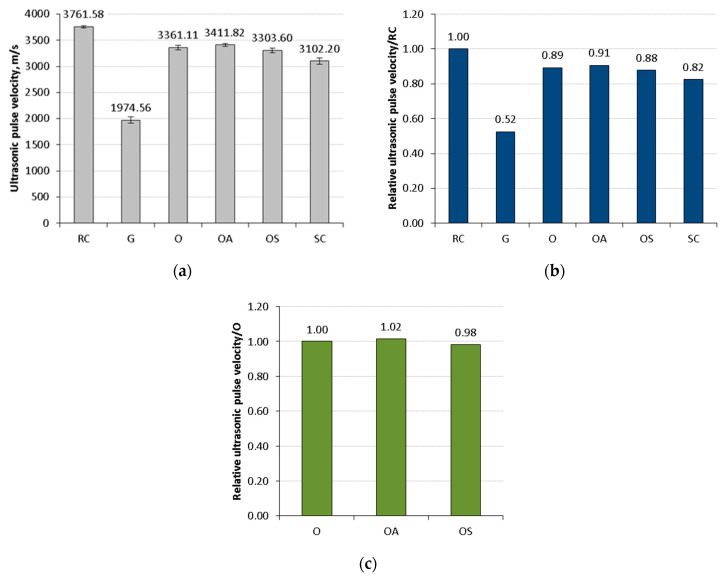
Ultrasonic pulse velocity (UPV) of concrete specimens: (**a**) absolute values; (**b**) relative values/RC; (**c**) relative values/O.

**Figure 10:**
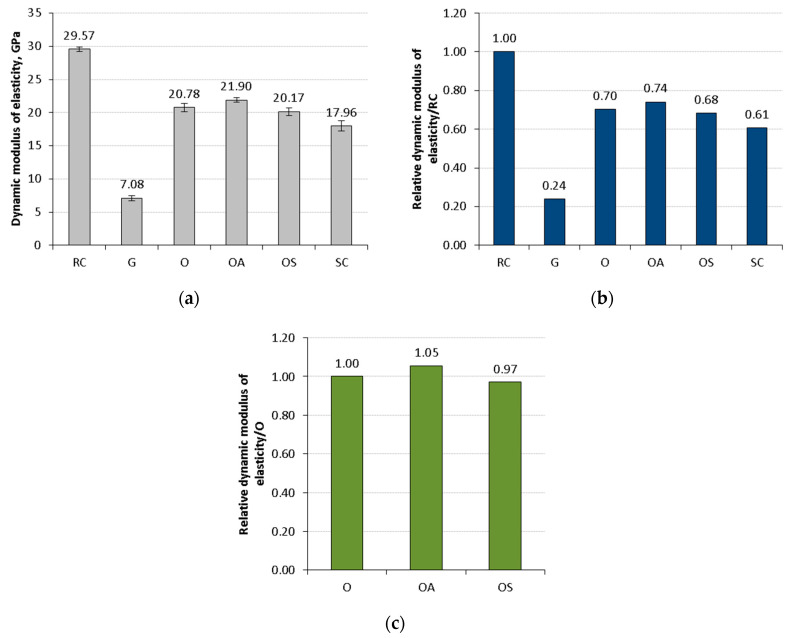
Dynamic modulus of elasticity of concrete specimens: (**a**) absolute values; (**b**) relative values/RC; (**c**) relative values/O.

**Figure 11:**
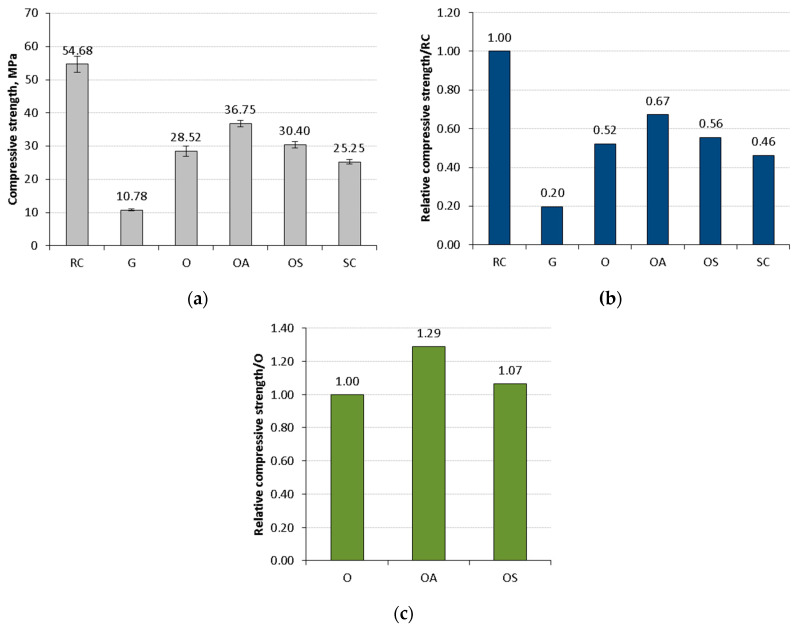
Compressive strength of concrete specimens: (**a**) absolute values; (**b**) relative values/RC; (**c**) relative values/O.

**Figure 12:**
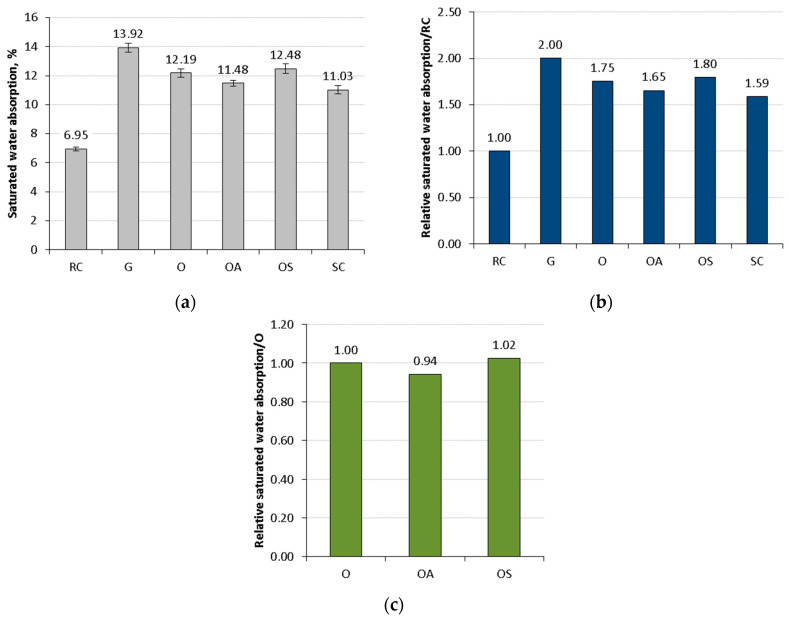
Saturated water absorption of concrete specimens: (**a**) absolute values; (**b**) relative values/RC; (**c**) relative values/O.

**Figure 13:**
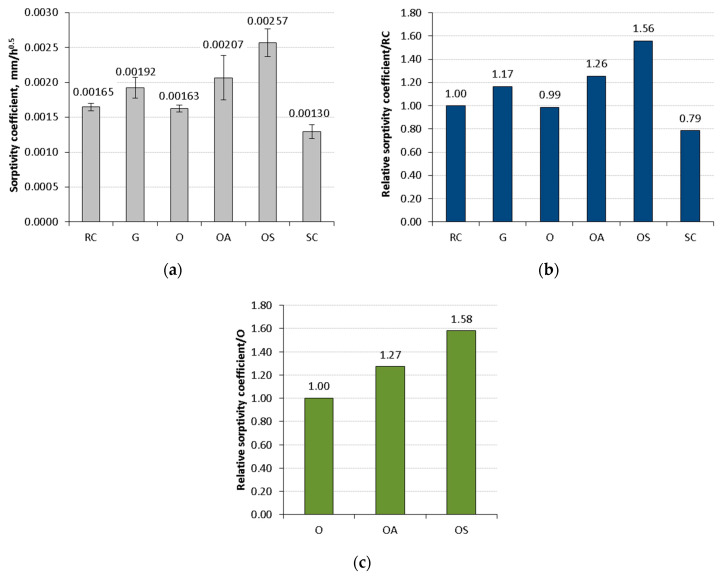
Sorptivity coefficient of concrete specimens: (**a**) absolute values; (**b**) relative values/RC; (**c**) relative values/O.

For the remaining specimens, the density, ultrasonic pulse velocity (UPV), dynamic modulus of elasticity, compressive strength, saturated water absorption and sorptivity coefficient results are presented in Figure 8, Figure 9, Figure 10, Figure 11, Figure 12 and Figure 13. Figures marked with (a) show the absolute values of measured values, (b) the relative values express the ratio between the mean measured results of each mixture and that of the reference concrete (RC) and (c) the relative values express the ratio between the mean measured results of mixture with ground olive pits and that of the mixture with untreated ground olive pits (O). The absolute values represent the mean of three measurements and include the standard deviation.

According to Figure 8a, the reference concrete has a density of 2322.19 kg/m^3^, while the density of concrete with partial replacement of agro-aggregates is 10 to 13% lower than the reference concrete (Figure 8b). All values are above 2000 kg/m^3^, which places all concretes in the class of normal or ordinary concretes. The ground olive pits treatment with ash water slightly increased the density of the concrete, while the sea treatment did not affect the obtained values (Figure 8c).

**Figure 14:**
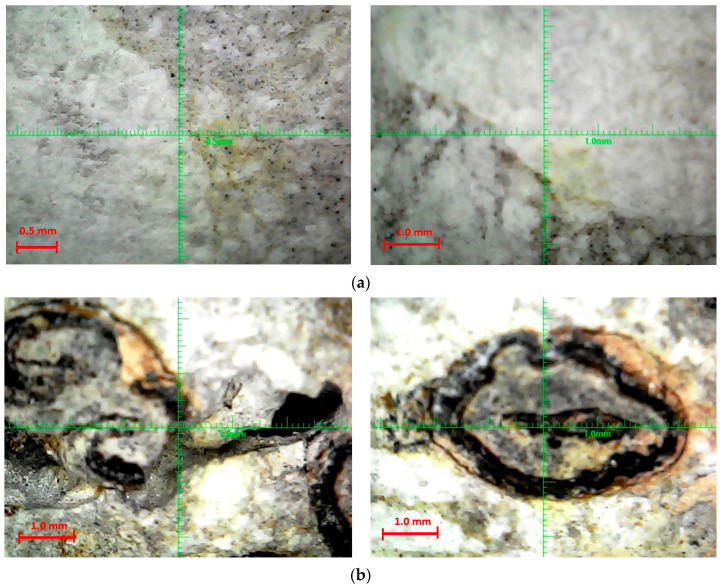

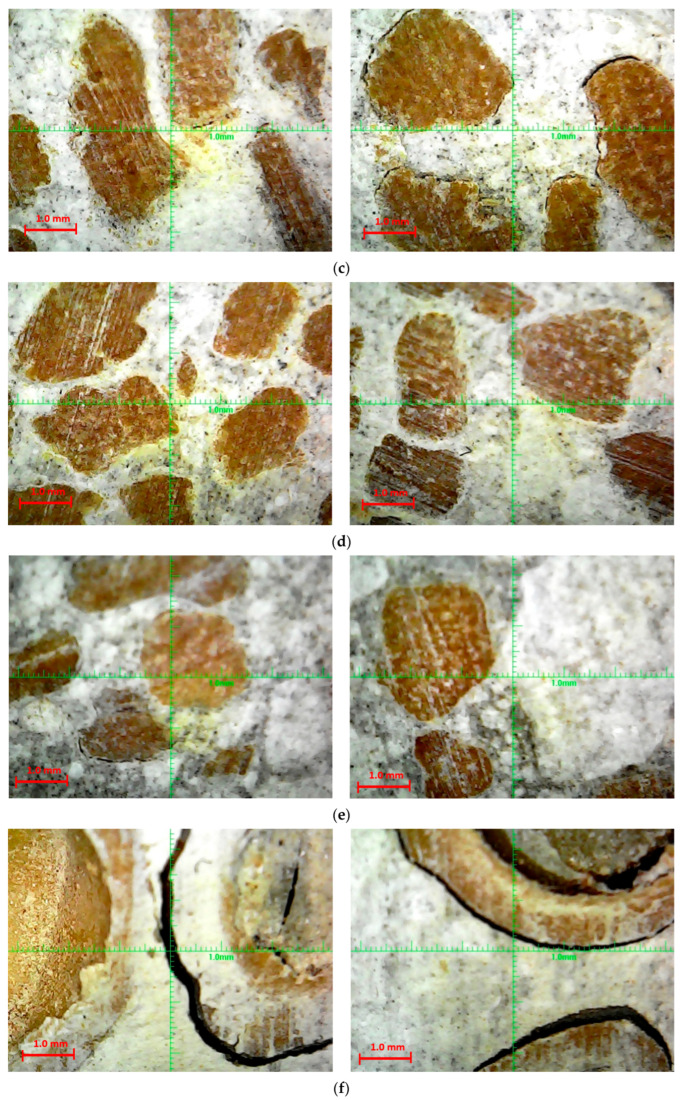
Microscopic image of concrete specimens: (**a**) RC: (**b**) G; (**c**) O; (**d**) OA; (**e**) OS; (**f**) SC.

Regarding the UPV, good concrete typically exhibits values in the range from 3600 to 4500 m/s [103]. Based on this criterion, RC is considered as good concrete, O, OA, OS and CH are questionable concretes, and G is poor concrete because it has a UPV value below 2100 m/s [103]; see Figure 9a. According to [104,105], all concretes with agro-aggregate belong to medium quality concretes (3000–3500 m/s), except for G, which is classified as doubtful concrete (2000–3000 m/s). Replacing part of the fine aggregate with grape seeds reduced the UPV value by 48%, while the other mixtures were in the range of 82–91% of referent concrete; Figure 9b. According to Kumar [66], higher reduction occurs due to increased voids, micro-cracks and defective aggregate–matrix transitions, while for concretes with UPV below 3200 m/s, as is the case with SC and especially G, the reason is poor internal compactness from high absorption and low aggregate strength. Figure 14 shows two optical microscope images of each specimen. In Figure 14b,f, the weak interconnection of agro aggregates and paste is clearly visible, i.e., the weak inter-surface transition zone (ITZ). Inside the grape seeds and cherry pits themselves, cavities and inhomogeneity are also visible. The situation is somewhat better with specimens from ground olive pits (O, Figure 14c and OS, Figure 14e), and the highest quality ITZ is visible on samples of reference concrete (RC, Figure 14a) and ground olive pits treated with ash water (OA, Figure 14d). Therefore, it is understandable that the highest UPV result of agro-concretes was obtained with the OA mixture, and the pre-treatment of ground olive pits with ash slightly improved the UPV of the concrete; Figure 9c. A similar trend was observed for the dynamic modulus of elasticity (Figure 10a), except that lower values were achieved compared to UPV, so that mixture G achieved only 24% of the value of the reference mixture, SC 61%, while the mixtures with ground olive stones ranged from 68% to 74%; Figure 10b. Seawater treatment reduced the value of the dynamic modulus of elasticity by 3%, while ash treatment increased it by 5% compared to mixture O; Figure 10c. The obtained values are in accordance with Figure 14.

Compressive strengths ranging from 10.78 MPa (G) to 54.68 MPa (RC) were achieved; Figure 11a. The replacement of a part of the aggregate had a significant impact on the results of compressive strength. The decrease in all compressive strengths can be ascribed to the inferior strength of agro-aggregates in comparison to that of conventional aggregates such as crushed limestone aggregates. According to [63], which reviews the properties of concrete with OPS aggregate, the reduction in compressive strength is associated with a weaker interfacial bond between OPS and the cement paste, as well as the increased porosity and water absorption of the OPS aggregate. According to Figure 14 and Table 1, this is particularly true for grape seeds and sour cherry pits. The reduction in compressive strength is the largest in mix G; replacing 30% of the fine aggregate reduced the strength by 80% compared to the reference concrete, as indicated in Figure 11b. In a study [67] where the fine aggregate was completely replaced with grape seeds, the strength decreased by 90% (6.23 MPa), so the result obtained is in accordance with that research; see Figure 14b and Table 1. Specimens with sour cherry pits achieved a strength of about 28.5% more than in the aforementioned study [67], but closer to the value in [73]. Compared to the reference concrete in this study, SC achieved only 46% of the compressive strength. Treatment of ground olive pits with ash water (OA) improved compressive strength by 29% compared to untreated specimens (O); Figure 11c. The OA mixture achieved 67% of the compressive strength of the reference concrete, which is in accordance with the research [74], although mortars were used there. The pre-treatment of agro aggregates has a significant effect on compressive strength, as shown in [104], where lime treatment of OPS aggregates contributed to an increase in compressive strength by about 8%. Yew et al. [45] indicated that the application of heat treatment improved the surface quality of OPS aggregates, which in turn enhanced the adhesion between the aggregates and the cement paste, resulting in a compressive strength of 49 MPa at 28 days and 52 MPa at 90 days.

If the water absorption is less than 5%, the concrete is deemed to be of high quality [106], but other sources indicate that a water absorption rate of concrete below 10% is classified as low [107,108]. Figure 12a shows that only referent concrete had water absorption under 10%. Agro-concretes have an increase in absorption of 59 to 100% compared to RC; Figure 12b. Such an increase can be attributed to the high water-absorbing characteristics of agro-aggregates compared to crushed limestone aggregates, as shown in Table 1. SC specimens have less water absorption than specimens with ground olive pits (O, OA and OS). Taking the explanation from [109] for behavior of crushed and original OPS aggregate, it could also be applied here: this could be attributed to the jagged and spiky edges of the crushed ground olive pits, which results in the crushed aggregates having a tendency to absorb water. In contrast, the surface texture of the convex faces of the sour cherry pits is relatively smooth, potentially leading to a decrease in water absorption. High increases in agro-concretes have also been measured by some other authors: for coconut shell aggregate concrete, Gunasekaran et al. [110] reported that water absorption was 10.66–11%, while Abutaha et al. [111] reported that when replacing coarse aggregate with oil palm boiler clinker in the range of 20 to 100%, there was an increase in water absorption in the range of 35% to 80%. Treatment of ground olive pits with ash water reduced absorption by 6%, while treatment with seawater increased it by 2% compared to untreated pits; Figure 12c.

The sorptivity coefficient of the concrete specimens is shown in Figure 13. The O and CH mixtures achieved even lower values compared to the reference concrete; Figure 13a,b. Bailo et al. [112] conducted a study on the sorptivity of concrete incorporating coconut shell. The decrease in water absorption observed with increased coconut shell content was likely due to disruption of capillary continuity within the concrete matrix. The authors attributed this to the more irregular and less permeable nature of the coconut shell particles, which creates barriers that impede fluid movement. This is the only property of concrete in which the ash water treatment had an adverse effect (Figure 13c), but it can also be seen that there is a large standard deviation in the individual results; Figure 13a.

**Figure 15:**
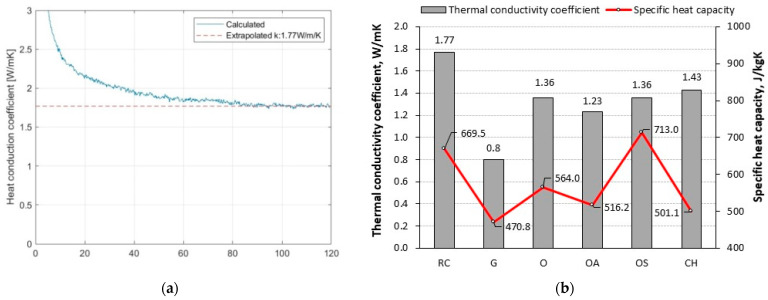
(**a**) Thermal conductivity coefficient (W/mK) vs. time (min) for RC; (**b**) thermal conductivity coefficient (W/mK) and specific heat capacity (J/kgK) for all specimens.

**Figure 16:**
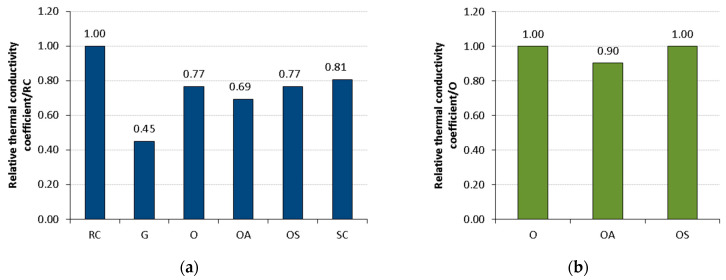
Relative thermal conductivity coefficient: (**a**) relative values/RC; (**b**) relative values/O.

The thermal conductivity coefficients are presented in Figure 15a,b. Figure 15a, created using MATLAB, illustrates an example of the extrapolated and calculated thermal conductivity coefficient, in this case for RC. Figure 15b displays the thermal conductivity coefficients obtained for all types of concrete, along with the corresponding specific heat capacity values. Additionally, Figure 16 highlights the relative thermal conductivity coefficients in comparison to the reference concrete, as well as the relative values for the mixtures containing olives.

The values for specific heat capacity follow the obtained thermal conductivity coefficient, except for the OS mixture. Since the value of specific heat capacity is affected by moisture, it is possible that the treatment of the aggregate with seawater increased the hygroscopicity of the concrete, but the phenomenon should definitely be investigated in more detail. The thermal conductivity coefficient of the reference concrete is 1.77 W/mK, which is expected for normal weight concrete, while the values for agro-concrete are slightly lower; Figure 15b. By replacing natural aggregate with agro-aggregate in the amount of 30% of the total aggregate volume, the reduction in thermal conductivity coefficients is from 19.2 to 54.8% compared to the reference concrete; Figure 16a. The lowest value was obtained for mixture G, with a thermal conductivity of 0.7997 W/mK (Figure 15b). For comparison, in study [67], a mixture with untreated grape seeds reached a value of 0.36 W/mK, corresponding to 25% of the reference concrete value (1.42 W/mK). In that study, the amount of grape seeds used in 1 m^3^ of concrete was 2.35 times higher than in the present study, which is consistent with the ratio of the resulting thermal conductivity values. A more comparable case is reported in [113], where the authors investigated the effects of replacing 20% of the total aggregate volume with agricultural waste-based capsules made from grape seeds and cherry pits, and found that the thermal conductivity was reduced by 32% and 22%, respectively. The mixtures with untreated cherry pits in the study [67] and here in the present study have almost the same composition, but different thermal conductivity coefficients: 0.70 W/mK and 1.43 W/mK, respectively. As previously noted, they also differ in compressive strength. Mixtures with ground olive pits have a higher reduction in the thermal conductivity coefficient and, again, the best mixture is OA. Treatment with ash water reduced the thermal conductivity coefficient by 10%; Figure 16b.

Based on all the above results, Table 4 was created, which shows the possible applications of the tested concretes as well as their practical potential. Table 4 is made according to [114,115], where concrete class C25/30 is the minimum standard for most structural elements in European construction. This class is widely utilized and is integral to various construction projects, especially in reinforced concrete structures [114]. While the O specimens also exhibit a mean value exceeding 30 MPa, this study, following EN 206 guidelines [100] and the number of available specimens, identifies only the OA specimen as meeting the required criteria. To positively evaluate other specimens containing ground olive pits, a significantly larger series of tests would be necessary.

## 5. Conclusions

This study investigated the influence of different agro-based aggregates (ground olive pits, grape seeds, sour cherry pits, and carob seeds) on the physical, mechanical, and thermal properties of concrete at 28 days. The results demonstrate that replacing 30% of natural fine aggregate with agro-aggregates significantly affects concrete performance. The most severe deterioration was observed in mixtures containing grape seeds (G), where compressive strength decreased by approximately 80% compared to the reference concrete (RC), accompanied by very low ultrasonic pulse velocity (UPV) and dynamic modulus of elasticity. This reduction is attributed to poor internal compactness, a weak interfacial transition zone (ITZ), high porosity, and inferior mechanical properties of the agro-aggregates.

Mixtures containing sour cherry pits (SC) also showed a considerable reduction in compressive strength (to approximately 46% of RC) and increased water absorption, confirming weak bonding between the aggregate and cement paste. Concrete containing ground olive pits performed notably better than other agro-concretes, particularly when the aggregates were pre-treated with ash water (OA). The ash treatment improved the ITZ quality, increased compressive strength by approximately 29% compared to untreated olive pits (O), enhanced dynamic modulus of elasticity, and slightly reduced water absorption. Among all agro-based concretes, OA exhibited the most balanced performance. All agro-concretes showed increased water absorption compared to the reference concrete, which may limit their durability in aggressive or moisture-exposed environments. However, a significant reduction in thermal conductivity (19–55%) was achieved in all mixtures, indicating strong potential for improved thermal insulation performance. The lowest thermal conductivity was observed in the grape seed mixture, although this was accompanied by poor mechanical performance.

Carob-based concretes (HC and WC) disintegrated during water curing due to seed swelling and tannin-related degradation, indicating unsuitability for structural or long-term construction applications without prior stabilization treatment. Overall, agro-aggregate concretes cannot replace conventional structural concrete in load-bearing applications at the tested replacement ratio. However, treated ground olive pits (OA) show promising potential for non-structural elements where moderate mechanical performance and improved thermal insulation are required.

Future research should focus on optimizing the replacement ratio of agro-aggregates in order to achieve a better balance between mechanical performance and thermal efficiency, particularly by investigating lower substitution levels (10–20%). The optimum concentration of ash in the ash water should be found, in addition to the duration of the treatment. Additional surface pre-treatment methods such as lime treatment, heat treatment, silicate impregnation, or polymer coatings should be explored to improve the interfacial transition zone and reduce water absorption. Long-term durability testing, including freeze–thaw resistance, sulphate attack, chloride penetration, carbonation, shrinkage, and creep, is necessary to assess service-life performance. Further microstructural investigations using SEM and porosimetry would provide deeper insight into pore structure development and bonding mechanisms. Moreover, the incorporation of supplementary cementitious materials (e.g., fly ash, slag, silica fume) should be evaluated to mitigate strength reduction and enhance durability. Finally, comprehensive thermal, acoustic, fire-resistance, and life cycle assessments are recommended to determine the full sustainability potential and practical applicability of agro-aggregate concretes in energy-efficient construction.

## Figures and Tables

**Table 1:** The properties and appearance of aggregates.

Type	Appearance	Density (g/cm^3^)	Bulk Density (g/cm^3^)	Weight Per Seed (mg)	Length (mm)	Width (mm)	Absorption%
Crushed limestone aggregate 0/4 mm	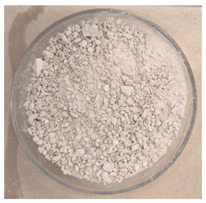	2.69	1.62	-	-	-	2.56
Crushed limestone aggregate 4/8 mm	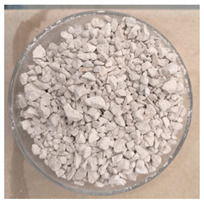	2.69	1.35	-	-	-	2.08
Grape seed	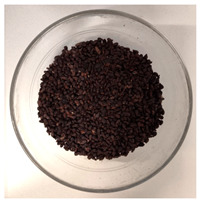	1.10	0.62 ± 0.01	27.97 ± 0.46	6.0 ± 0.4	3.0 ± 0.4	53.3
Sour cherry pit	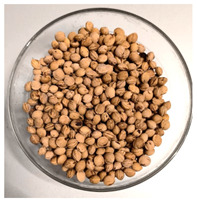	0.81	0.48 ± 0.01	219.74 ± 0.32	8.6 ± 0.7	7.6 ± 0.5	36.4
Untreated ground olive pit	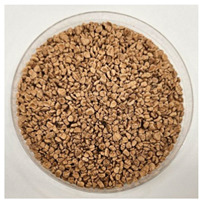	1.05	0.703 ± 0.008	-	-	-	28.7
Ground olive pit treated with ash water	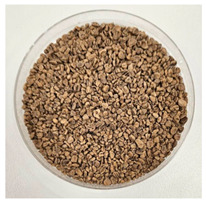		0.693 ± 0.010	-	-	-	28.6
Ground olive pit treated with seawater	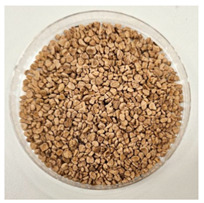		0.702 ± 0.025	-	-	-	29.8
Whole carob seed	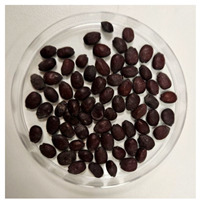	1.36	0.812 ± 0.024	191.69 ± 34.33	9.7 ± 0.8	6.0 ± 0.8	
Hulled carob seeds	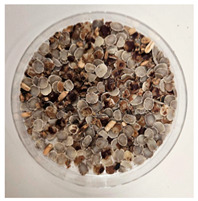		0.704 ± 0.115	46.99 ± 2.16	7.0 ± 0.0	5.0 ± 0.0	

**Table 2:** Composition for 1 m^3^ of concrete.

Components		Mixtures	RC	G	O	OA	OS	SC	WC	HC
Cement, kg			400	400	400	400	400	400	400	400
Water, kg; *w*/*c* = 0.45			180	180	180	180	180	180	180	180
Aggregate, kg	Crushed limestone	0/4 mm	1239.4	708.2	708.2	708.2	708.2	1239.4	1150.9	619.7
		4/8 mm	531.2	531.2	531.2	531.2	531.2	-	265.6	619.7
	Grape seed		-	217.2	-	-	-	-	-	-
	Sour cherry pit		-	-	-	-	-	160.5	-	-
	Ground olive pit	untreated	-	-	207.3	-	-	-	-	-
		ash water	-	-	-	207.3	-	-	-	-
		seawater	-	-	-	-	207.3	-	-	-
	Carob seed	hulled	-	-	-	-	-	-	-	268.6
		whole	-	-	-	-	-	-	179.0	-
Superplasticizer, kg			2	2	2	2	2	2	2	2

**Table 3:** Concrete workability and standardized consistence classes EN 206.

Concrete	RC	G	O	OA	OS	SC	WC	HC
Slump, mm	50	0	255	225	0	95	20	0
Standardized consistence classes	S2	S1	S5	S5	S1	S3	S1	S1

**Table 4:** Possible application of concrete with fruit pits and seeds.

Mix	Compressive Strength, MPa	Structural Application	Non-Load Bearing Elements	Insulation Use	Practical Potential
RC (Reference Concrete)	54.7	Yes	Yes	No	Very High
OA (Ground Olive Pits–Ash Treated)	36.8	Limited	Yes	Yes	Highest among agro-concretes
O/OS (Untreated/Seawater Treated Olive Pits)	28.5, 30.4	No	Yes	Yes	Moderate
SC (Sour Cherry Pits)	25.3	No	Limited	Limited	Low
G (Grape Seeds)	10.8	No	Limited	Yes	Mainly as insulating material
HC/WC (Carob–Hulled/Whole)	-	No	No	No	Not suitable for construction

## Data Availability

The original contributions presented in this study are included in the article. Further inquiries can be directed to the corresponding author.
